# Health worker perspectives on user fee removal in Zambia

**DOI:** 10.1186/1478-4491-10-40

**Published:** 2012-10-30

**Authors:** Barbara S Carasso, Mylene Lagarde, Caesar Cheelo, Collins Chansa, Natasha Palmer

**Affiliations:** 1 London School of Hygiene & Tropical Medicine, 15-17 Tavistock Place, London, WC1H 9SH, UK; 2 University of Zambia, Lusaka, Zambia; 3 Ministry of Health, Lusaka, Zambia

**Keywords:** User fees, Motivation, Human resources, Health financing, Free care, Zambia

## Abstract

**Background:**

User fees for primary care services were removed in rural districts in Zambia in 2006. Experience from other countries has suggested that health workers play a key role in determining the success of a fee removal policy, but also find the implementation of such a policy challenging. The policy was introduced against a backdrop of a major shortage in qualified health staff.

**Methods:**

As part of a larger study on the experience and effect of user fee removal in Zambia, a number of case studies at the facility level were conducted. As part of these, quantitative and qualitative data were collected to evaluate health workers’ satisfaction and experiences in charging and non-charging facilities.

**Results:**

Our findings show that health-care workers have mixed feelings about the policy change and its consequences. We found some evidence that personnel motivation was higher in non-charging facilities compared to facilities still charging. Yet it is unclear whether this effect was due to differences in the user fee policy or to the fact that a lot of staff interviewed in non-charging facilities were working in mission facilities, where we found a significantly higher motivation. Health workers expressed satisfaction with an apparent increase in the number of patients visiting the facilities and the removal of a deterring factor for many needy patients, but also complained about an increased workload. Furthermore, working conditions were said to have worsened, which staff felt was linked to the absence of additional resources to deal with the increased demand or replace the loss of revenue generated by fees.

**Conclusion:**

These findings highlight the need to pay attention to supply-side measures when removing demand-side barriers such as user fees and in particular to be concerned about the burden that increased demand can place on already over-stretched health workers.

## Background

User fee policies have been at the centre of debates on health financing since several international actors pushed for their introduction in the 1980s [1]. Recently, most of the attention has focussed on the negative consequences of user charges on equitable access to health care [2], and there is a growing consensus favouring the abolition of user fees for essential health care services [3]. Yet, some have underlined the potential pitfalls of removing fees without adequate planning [4], and there is limited evidence suggesting that abolishing user charges can increase utilisation at the expense of quality of services [5]. The involvement and motivation of health-care workers, or usually the lack thereof, have been underlined as key elements in the implementation of the policy change in several countries [6-8].

While there is a large array of factors potentially shaping health workers’ motivation [9,10], financial incentives, in particular higher remuneration, is systematically cited as one of the most important ones [11-14]. Health user fees were introduced in Zambia at the beginning of the 1990s ^a^, with improving staff motivation as one of the objectives. In 2006 they were again removed from government and mission ^b^ health centres and district hospitals in rural districts, a policy that was extended to cover peri-urban areas 1 year later. The policy decision was taken in view of the poverty levels in the country, the high cost for accessing health services, and the desire to provide universal access. This article forms part of a series documenting the experience of this change in user fee policy in Zambia [15-17], examining the perspectives of health workers on the change in policy.

The policy change was introduced against a background of a shortage of health workers in Zambia, which was particularly severe in rural areas [18]. According to the Human Resources for Health Strategic Plan 2006–10, more than 50% of rural health centres had only one qualified staff member and numerous facilities were without any professional staff at all [19]. It was envisaged that additional health workers would be recruited in rural areas to respond to the anticipated increase in demand for services [20], but in practice this did not occur on a large scale.

The abolition of user fees can affect health workers in a number of ways. Assuming that user fee removal leads to higher utilisation, workload will increase if the number of health workers remains the same. Other inputs such as drugs will also be put under pressure unless more resources are made available. Health workers may feel that the quality of care that they can deliver is compromised by these additional pressures. Furthermore, fees may have been used to provide incentives to staff, and so removal of fees can mean a direct financial loss for them. Health workers will also have their own perspective on the desirability of the policy change and the degree to which new patients seeking care are in genuine need.

Despite several countries having removed fees in the last 2 decades, there is little detailed evidence about how fee abolition has affected the morale of health staff. One study from Uganda suggests that health workers felt they were not adequately motivated given the increased workload after fee removal, despite an increase in salary [21]. Evidence from South Africa suggests that nurses were supportive of the broad principles of free care but complained about the implementation of the policy; they reported workload to be higher while conditions deteriorated, and patients were seen as ‘abusing’ free services [22]. Findings from Ghana show that an increase in attendance rates after the introduction of a fee exemption scheme did not negatively affect health worker morale. This was largely attributed to the increased and relatively high salary of health workers in the sector [23].

This study sought to explore the consequences for health workers of removing user charges in Zambia. This article has three specific objectives. First, it assesses whether workers in facilities where fees had been removed would be less satisfied with their work than those in charging facilities. Second, it explores the views of health workers on how the policy change has affected their working environment. Finally, it captures the views of health staff on the impact of the policy change for patients.

## Methods

### General context of the study

Zambia is divided into 9 provinces and 72 districts. In 2011, Zambia had one of the lowest scores on the Human Development Index for Southern Africa (0.430), ranking 164 in the world [24]. The health-care system in Zambia is faced with diverse challenges ranging from inequity in the provision of health services to poor quality of care and critical shortage of human resources. This situation is worsened by a high burden of communicable diseases. Zambia has one of the highest prevalence rates for HIV/AIDS in Africa (14%), and although the tuberculosis (TB) treatment success rate has improved in recent years, the TB notifications per 100,000 populations are still high (373 per 100,000 in 2010). Finally, maternal mortality is still very high, at 591 per 100,000 live births, off track to attain the Millennium Development Goal objective [25]. In addition to the services provided by the public sector, there is an emerging urban private-for-profit sector, some private mine-based hospitals, and a not-for-profit private sector working in close partnership with the public services.

### Selection of study sites

As part of a larger evaluation of several aspects of user fee removal in Zambia [15], data were collected between October and December 2008 in five districts, purposively selected from the 72 districts of the country. Two were rural (with fees removed) and three were urban districts (fees only removed in outlaying parts of the district). In each district, four facilities were chosen in consultation with the District Health Management Team (DHMT). These consisted of a combination of district hospitals and health centres, government- and mission-run facilities, located in rural, peri-urban, and urban areas. Selection criteria were based on accessibility during the data collection period and likely availability of health staff at the facility to provide the information that was required. A total of 20 health facilities were visited, of which 14 had removed fees and 6 were still charging fees.

### Data collection

In each facility, self-administered questionnaires were distributed to health-care providers, and in-depth interviews were conducted with at least one and at most five staff members (see final sample details in Table 1). The questionnaires and interviews were all done in English.

**Table 1:** Final sample characteristics, per type of facility

	**Facilities visited**		**Self-administered questionnaire (quantitative section)**		**Self-administered questionnaire (qualitative section)**		**Key informant interviews**	
	***Non-charging***	***UF charging***	***Non-charging***	***UF charging***	***Non-charging***	***UF charging***	***Non-charging***	***UF charging***
**Total**	**14**	**6**	**57**	**33**	**52**	**0**	**37**	**16**
*Of which:*								
**Mission facility**	7	0	33	0	30	0	21	0
**Government facility**	7	6	24	33	22	0	16	16
*Of which:*								
**District hospital**	4	1	26	9	24	0	13	3
**Health centre**	10	5	31	24	28	0	24	13

UF = user fee.

In the smaller health centres with few staff members, everyone was invited to fill out a questionnaire. In the larger facilities and district hospitals, questionnaires were distributed to personnel representing the different departments and wards.

In addition to questions capturing their basic descriptive characteristics (health worker cadre, gender), the self-administered questionnaire contained questions where respondents had to give their level of agreement or satisfaction (on a 5-point Likert scale) with a series of statements relating to different dimensions of motivation (quantitative section, see Table 2). This short list of statements was constructed for the purposes of this study based on Hertzberg’s two-factor motivation theory and its distinction between motivating and hygiene factors [26]. The mix of statements allows health workers’ perceptions of various characteristics of the working environment whose absence or inadequate level (e.g. pay, workload) can yield dissatisfaction (hygiene factors) as well as intrinsic aspects of the job whose existence (e.g. recognition, responsibility, opportunities) provide positive satisfaction (motivating factors). The list of statements also reflects some of the traditional factors cited by health workers in low-income countries as linked to (de)motivation [10,13]. Finally, a pilot study helped us ensure that the wording of the statements made sense in the Zambian context.

**Table 2:** Likert scale statements used to assess domains of motivation in the questionnaire

**Ref**	**Please indicate how satisfied you are with each of the following aspects of your job by ticking the appropriate box:**	**Very satisfied**	**Satisfied**	**Neither satisfied nor dissatisfied**	**Dis-satisfied**	**Very dissatisfied**
1	Your colleagues and fellow workers					
2	Your workload					
3	Recognition you get for good work					
4	Amount of responsibility you are given					
5	Opportunities for in-service training					
6	Your salary					
7	Your hours of work					
8	The amount of administrative tasks/ paperwork					
9	Taking everything in consideration, how do you feel about your job?					
**Ref**	**For each of the following statements, please say how much you agree or disagree:**	**Strongly disagree**	**Disagree**	**Neither agree nor disagree**	**Agree**	**Strongly agree**
10	There are too many patients to attend to					
11	Patients are too demanding					
12	Medical supplies are usually available					
13	I intend to quit this job soon					
14	I have not enough time to do my job well					
15	There are usually enough drugs in this facility					
16	I would leave my job to work for the private sector if I got an opportunity					

The questionnaire concluded with two open-ended questions, which asked respondents to describe how the removal of user fees had changed their situation as a health worker, and that of the patients. This part of the questionnaire was only distributed to staff working in non-charging facilities.

At each facility, in-depth interviews were conducted with senior or longer-serving staff, mostly the facility managers. General questions were asked to gather background information on the following: the facility, implementation of the user fee policy (for facilities that removed fees), trend in utilisation rates, revenue from user fees, drug management, and overall perception of the impact of the policy change. Furthermore, their perception on issues affecting the different cadres of health workers in the context of the abolition of user fees were documented. To avoid inhibiting respondents unfamiliar with such practices, it was decided that key informant interviews would not be recorded. Instead, the researchers took notes and wrote down illustrative quotes during the interview.

At each facility visited, routine data were also collected on trends in outpatient visits, staff levels, drug availability, and facility financing over time. Selected findings are reported to describe the context in which the health workers operated. More detailed analyses of these other features of the facilities have been reported elsewhere [27,28].

### Quantitative analysis

Using STATA, principal component analysis (PCA) was used to analyse participants’ responses to specific questions on motivation, measured through various constructs. This type of analysis is used to reduce the information contained in a large number of questions to a few variables (called principal components) that will capture underlying themes reflected in those questions. After running a first PCA with all variables, we identified a few groups of variables that seemed to explain similar dimensions of motivation. We then ran separate PCAs with these different groups of variables and created an index variable “summarising” these different variables by taking the first component of each PCA. Each index therefore reflects how respondents feel about three different constructs influencing their motivation. The correlation of underlying variance between variables identified in the three groups was ascertained using Cronbach’s alpha. This measure of the internal consistency between several variables, comprised between zero and one, indicates the degree to which a set of items measures a single unidimensional latent construct. A Cronbach’s alpha greater than 0.6 typically indicates a good overall consistency of the different variables, which can therefore be assumed to measure the same construct.

To test whether there were differences between staff’s motivation in facilities that are charging vs. non-charging, indexes were calculated for each of the sub-populations and a *T*-test was performed to test for the statistical significance of differences observed. In addition, differences in responses between staff working in district hospital vs. health centres and government vs. mission facilities were also assessed.

### Qualitative analysis

Responses to open-ended questions in self-administered questionnaire and notes from key informant interviews were coded, grouped thematically, and analysed using content analysis. Themes were ranked according to how often they appeared in responses, and representative excerpts from what health workers wrote down in this part of the questionnaire were selected to illustrate the themes.

## Results and discussion

### Who were the health workers?

Table 3 gives some details of the characteristics of the health workers who filled out the questionnaire. As is common in primary level facilities in an area suffering from shortages of human resources, there was a wide variety of qualifications, although it is to be expected that these different cadres were fulfilling similar functions. Health workers in charging and non-charging facilities were comparable in terms of years of work at the health facility and reported number of working hours, which were estimated at 8 h per day.

**Table 3:** Respondents of the self-administered questionnaire

	**Non-charging facilities**	UF charging facilities*
Female:male	29:28	27:6
Nurses	22	12
Midwives	4	8
Pharmacy dispensers	7	3
Classified daily employees	8	2
Clinical officers	4	4
Time worked in facility (median [range])	3.5 years [0.1 – 30.9]	3.2 years [0.1 – 24.9]

UF = user fee; *the qualitative section of the questionnaire was only filled out by staff in non-charging facilities.

### Contextual factors

Utilisation data collected at the 20 case study facilities were of poor quality, which prevented us from matching reliable quantified information to health workers’ perceptions at a facility level. However, data on utilisation collected and analysed for the broader study [17] suggest that following fee removal, overall outpatient visits increased in rural districts. Utilisation data collected for the broader study from the national Health Management Information System were aggregated by district and could therefore not be matched to the facilities visited. However, a wide difference across districts was observed, and the increase was not always sustained over time. Our analysis also suggested that the number of qualified staff employed in the case study facilities decreased after fee removal. These findings would imply that in objective terms, workload of health workers has increased since the policy change.

Ten per cent of the revenue raised from user fees was used as salary top ups in some districts visited, whereas the rest was used to purchase basic material for the health facility, to hire additional staff (classified daily employees, CDEs), or to finance community activities. Managers confirmed that the provision of health services relied heavily on the support of CDEs, especially in rural and remote areas. Fee removal therefore compromised the financial means available to hire CDEs and improve the working environment.

Moreover, the year of the policy change was characterised by a 40% drop in funding to districts and facilities as well as widespread drug shortages, attributed to wider health systems issues [29]. This situation further challenged the environment in which the health workers had to provide care.

### Self-reported motivation

The PCA analysis led to the identification of three groups of statements that capture opinions about similar constructs. These relate to (1) satisfaction with the volume of work, (2) extrinsic motivation (driven by external factors such as rewards or others’ opinions ^c^), and (3) satisfaction related to working conditions (see table 4).

**Table 4:** Key underlying themes of motivation identified from self-administered questionnaire

**Theme**	**Domains included (see Table 2)**	**Cronbach’s alpha**
**Satisfaction with volume of work**	2, 7, 10, 14	0.6477
**Extrinsic motivation**	3, 6, 16	0.6733
**Satisfaction with working conditions**	1, 3, 4, 5, 12, 15	0.6483

**Figure 1:**
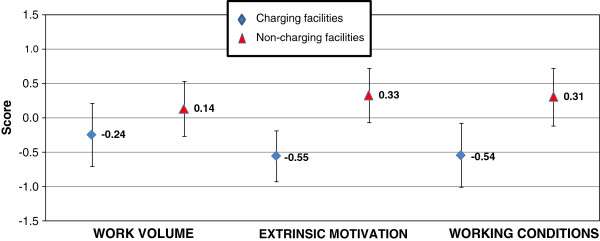
Staff responses for three main components of job satisfaction - charging compared to non-charging facilities.

Figure 1 depicts the average scores of satisfaction for the three main constructs identified. Contrary to expectations, staff working in non-charging facilities appears more satisfied than their colleagues working in charging facilities on all three dimensions. Overall, no statistically significant difference in the level of satisfaction with regards to workload could be detected. However, staff in non-charging facilities indicated being more sensitive to reward-driven motivational factors (*p* = 0.0032) and were more positive about their working conditions (*p* = 0.0011).

**Figure 2:**
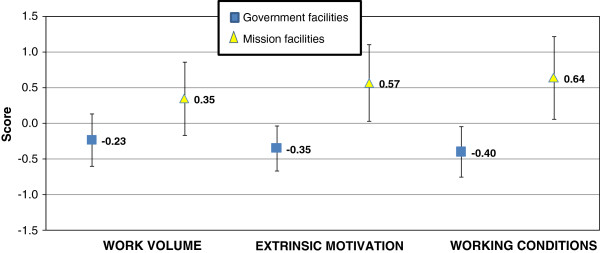
Staff responses for three main components of job satisfaction - government compared to mission facilities.

No difference was found between the responses of staff working in district hospitals and health centres. However, when comparing the level of satisfaction between government and mission facilities, there was evidence that mission staff were more satisfied on all three dimensions than staff working in government facilities (*p* = 0.0595, *p* = 0.0020, *p* = 0.0014 respectively; see Figure 2). Mission facilities are typically located in rural non-charging areas, which is likely to have inflated satisfaction levels in this group. If staff working in mission facilities is excluded from the analysis, the difference between charging and non-charging (government) facilities disappears.

### Health workers’ perceptions on user fee removal

In response to the two open-ended questions on how the removal of user fees has changed their personal situation as a health worker, and that of patients, many staff wrote long accounts of their experiences. Table 5 cites the most frequently raised issues, which are illustrated below with representative excerpts written down by the health workers.

**Table 5:** Issues (frequency) mentioned by staff about how fee removal has changed their personal and patients’ situations

**Changed personal situation**	**Changed situation for patients**
Higher workload (23)	Poorest patients can now access care (32)
Fewer resources for essentials in facility (11)	Patients come with minor complaints (14)
More drug shortages (6)	Worse for patients as drug shortages (2)
Higher job satisfaction (6)	Longer waiting time (1)
No more top-ups/bonus (3)	

### How free care changed the working environment of health workers

The issue health workers raised most often was a significant increase in workload. Some staff reported that this included being called to see patients outside working hours and having “*no more time for lunch breaks as there are too many people waiting*”.

"“User fee removal has increased the workload for me that patients can come in any time of the hours and you are forced to attend to them. Hence as a worker when fully fatigued you stop concentrating on your work properly; rather due to fatigue attitude towards the patients has become very bad”. Nurse, rural health centre (self-administered questionnaire)"

"“I am trained as an EHT [environmental health technician], but I do deliveries during the night and then there are day patients waiting which I attend to. I also work in the dispensary. But actually I am supposed to be in the community attending to problems there. I worked till 4 a.m. last night, and was here again at 7 a.m. Many patients are waiting, and there’s a woman in labour in the maternity ward. And it’s just me and a nurse”. Health worker, rural health centre (self-administered questionnaire)"

In seven facilities where the number of authorised positions was known, more than half of these posts were vacant. Facilities located in the most remote areas were found to be especially dependent on the help of classified daily employees (CDEs) to provide care. Some facilities had to lay off CDEs after the policy change as these salaries used to be paid for with user fee income.

"“We only have three qualified staff on the government payroll. An additional eight CDEs are employed with our ‘own’ (mission) money or through creative handling of the government grant. The problem for CDEs is the unpredictable payment of salary, which regularly comes months late”. In-charge, rural mission hospital (key informant interview)"

The second most cited issue from the questionnaire was that the policy change had worsened already challenging working conditions. This was because the income from fees was often seen as essential to purchase items like supplementary drugs, fuel for the ambulance and generator, cleaning material, stationary, or food for admitted patients.

Drug shortages, especially at the end of the month, were also reported by a number of staff, both as making their own job more difficult as well as impacting negatively on the patients who are then forced to “*go to the chemist and drugs are expensive there*”.

"“User fees were used to help us to get the necessary things e.g. supplementary drugs that are not found in the drug kits. How can I treat a patient when I cannot give him the right drugs? Also it has brought poverty in the health centre as the present grants sent to the health centre are not much to meet all the things that urgently need to be bought either for the community or health centre. Sometimes the grant comes too little or comes at a time when you have waited for too long”. M&E officer, rural health centre (self-administered questionnaire)"

"“User fee removal has not changed anything for the patients, instead it has worsened their suffering because of no medicine”. CDE, rural health centre (self-administered questionnaire)"

Respondents also wrote about positive experiences with the policy change, mainly from the point of view of the patients. Relating to how it had changed their own situation, a number of staff said that they got personal satisfaction out of the fact that fee removal enabled them to provide care to all, including the poorest of the community.

"“It has changed my personal situation due to that not every patient was able to pay and it’s not only the rich who needed treatment but everyone”. Medical doctor, rural hospital (self-administered questionnaire)"

"“The removal of user fees has really done me, personally as a health worker, good. Many times where most of the patients I see are from the remotest part of the county, who can’t even afford a meal. So, where on earth would they get money to pay at a health facility? I really thank government for scrapping it off. Many times when I would personally pay for a patient, looking at the condition because I can’t let them go back home sick”. Nurse, rural hospital (self-administered questionnaire)"

Staff also highlighted the discontinuation of salary top-ups since the policy change. In some districts, 10% of the income from user fees was used for top-ups on salary for the staff. Even though one nurse commented that the bonus for staff was negligible, others responded that it was crucial to help them cover the cost of transport ^d^.

"“…to my side as a worker it has been or it is very difficult for…things such as stationary and emergency transportation for patients. Health workers were getting incentives like transport allowances which were stopped with the removal of fees”. CDE, rural health centre (self-administered questionnaire)"

### How free care changed situation for patients

Half of the respondents said that fee removal has changed the patients’ situation for the better, as especially the poorest could now access care. For them, the “*fee used to be a significant – and sometimes insurmountable – barrier”*. They also noted down that fees caused people to postpone seeking care as “*some would come late the hospital fearing the charges yet they were very sick*”.

"“It has made it possible for patients to come to the hospital when there is need for them to be attended to. It has made the patients feel that they are considered and cared for even if they don’t have money – their health will be considered first”. Pharmacy dispenser, rural hospital (self-administered questionnaire)"

"“It has encouraged them to come and deliver from the health facility unlike in the past when they used to come when it was already too late when they see a serious complication”. Midwife, rural hospital (self-administered questionnaire)"

"“UFR has made it easy for patients who are poor to attend treatment at the right time. Some could come very late to the hospital fearing the charges yet they were very sick, sometimes they didn’t have that money to pay. Thanks for removal of user fees because not everyone could afford this”. Nurse, rural health centre (self-administered questionnaire)"

Less positively, health workers also wrote that fee removal had resulted in many patients coming forward with minor complaints; they “*just come and collect drugs”, “have stopped seeing the importance of health matters knowing that everything is free for them*”, or “*lose card numbers carelessly as they know they will not be charged for that”.*

In conclusion, health workers felt on balance that the removal of fees has made their own jobs more challenging, but also often more rewarding. Health workers were quite supportive of provision of free care, and it appears they would be more so if additional health staff could be deployed to cope with the higher workload and the income previously raised from fees could be compensated to buy essentials for the facility. As two health workers summed it up:

"“On the part of patients, it has encouraged them to come to the health centre whenever they feel unwell, unlike when they had to work for the user fee before they are attended to. On the other hand, the health centre is struggling in raising enough funds to run it because money from the government is not enough. I suggest if the government would increase and not the patients to pay”. Pharmacy dispenser, rural hospital (self-administered questionnaire)"

"“The removal of user fees has made the work to be good and enjoyable to me because everyone is able to manage to come. The only problem to me as a health worker there is too much work-overload as a result I cannot meet the demand of all the patients’ need. I therefore appeal that if the government can employ more health workers it can be better so that we can meet the needs of the patients”. CDE, rural health centre (self-administered questionnaire)"

The research described here attempted to investigate the ways in which health workers experienced the removal of user fees in Zambia. Our findings show that health care workers have mixed feelings about the policy change and its consequences. We found some evidence that health workers’ motivation was higher in non-charging facilities compared to facilities still charging. Yet it is unclear whether this effect was due to differences in user fee policy or to the fact that many staff members interviewed in non-charging facilities were working in mission facilities. To be able to tease out these two effects, future research should investigate whether there are systematic differences in the motivation of health workers in government and mission facilities where contextual elements are similar. At the same time, health workers expressed satisfaction at an apparent increased number of patients visiting the facilities and the removal of a deterring factor for many needy patients, but they also complained about their increased workload. Furthermore, working conditions were said to have worsened, which they linked to the absence of additional resources to deal with the increased demand or replace the loss of revenue generated by fees.

Some of the facilities in especially the most remote areas had very few qualified personnel. For instance, one rural health centre only had one volunteer present at the day of the visit who was so busy attending to patients that the research team did not want to keep him away from his work. This limited the amount of data that could be obtained, which means that the less staff a facility had, the less their views are represented in the sample of questionnaires. It is thus likely that the most overworked staff members are underrepresented.

This study was a cross-sectional survey and it is therefore not possible to know to what extent there was a difference between the two groups of facilities before the policy change. A survey done in 2006 (before fees were removed) showed that levels of staff satisfaction in rural (government and mission) health centres were higher than in urban health centres, with 56% of the rural health workers reporting to be ‘satisfied’ or ‘highly satisfied’ compared to 41% of staff working in urban health centres [30]. This would suggest that the differences between charging and non-charging facilities may have pre-dated the policy change.

It has been stated in previous work on user fee removal that health workers were sometimes hostile to the policy because of the challenges that it posed for their work [21,22,31,32]. Although this was not striking in the quantitative findings of our study, the qualitative part of the study showed that heath workers blamed the policy change for worsening working conditions. Health workers often stated that they were struggling with fewer resources to buy essentials for the facility, fewer drugs, and no more bonuses from fees. However, it is unclear whether these issues were indeed directly linked to the policy change, as the new policy was implemented during a period characterised by a drop in the district health budget, widespread drug supply problems, and a stagnating workforce in rural areas [15,33,34]. Frustrations relating to patients coming with minor complaints or just for the collection of drugs were also cited. However, this was difficult to match with actual cases, while there could have been alternative explanations for this phenomenon, for example, that people would come and seek care sooner in a less advanced stage of the disease and hence the conditions attended would become less severe.

On the other hand, we also found significant evidence that health workers derived utility from the feeling that ‘everyone can now access care’. Similar feelings were reported in Ghana, where health workers cited the smaller burden on the poor as an important benefit of the free care policy [23]. Overall, the health workers interviewed in Zambia appeared supportive of free care, as long as more health staff were deployed to cope with the higher workload and the income previously raised from fees was compensated for by government so that they could still buy essentials for the facility.

## Conclusions

Health workers are key implementers of any reform such as the provision of free care, and it is crucial to consider how they may support or thwart the implementation of such policy change.

Our results suggest that health workers felt motivated by the idea of working in a system that provides care free to those in need. They placed a value on benefits to patients and in particular greater equity in access to care.

Our findings also stress the need for adequate strategies to support health workers by providing additional resources for a policy change that is expected to increase utilisation and workload for staff. Failing to do so will worsen working conditions, triggering frustrations that are likely to override this positive predisposition.

Supportive strategies could include the recruitment of additional staff, payment of extra incentives, and the provision of extra drugs and substitute funding at the facility level.

These findings highlight the need to pay attention to supply-side measures when removing demand-side barriers such as user fees and in particular to be concerned about the burden that increased demand can place on already over-stretched health workers.

## Endnotes

^a^A flat fee was charged (which included payment for drugs), whereas children under 5 years, the elderly, and pregnant women were exempt.

^b^Mission facilities are part of the government system and their staffs are employed by the government. They are thus subject to national policy directives and civil service guidelines, but often receive additional support in terms of money or drugs from external sources. Virtually all mission facilities are located in rural areas and hence subject to the policy change.

^c^Extrinsic motivation is usually opposed to intrinsic motivation. An intrinsically motivated person will get pleasure or satisfaction from working on or completing a task. For example, an intrinsically motivated health care worker will feel rewarded and satisfied by the simple fact that she is taking care of patients. By contrast, an extrinsically motivated person may not necessarily enjoy taking care of patients, but she will do it because of the various external rewards (financial compensation, status, recognition from peers or the community, etc.) she gets for completing it.

^d^This part of the questionnaire was only given out to staff in rural areas; during key informant interviews it was suggested that the 10% top-up from fees was even more important for staff working in urban areas as the amount of revenue collected there is typically larger.

## Competing interests

The authors declare no conflict of interest.

## Authors’ contributions

BC drafted the proposal for the study, carried out the data collection, conducted the analysis, and took the lead in writing up the study. ML supported the tools for data collection, provided substantial input in the data analysis (especially PCA), and critically reviewed the manuscript. CChe provided input in the original design of the overall study assessing the impact of fee removal in Zambia, participated in the data collection, and helped with the write up of the study. CCha provided input in the original study design, facilitated data collection, and provided critical comments on the manuscript. NP is the PI of the study, conceived of the original study, participated in the HR proposal, provided input in data collection and analysis, and critically commented on the manuscript. All authors read and approved the final manuscript.
